# Seasonal Variation in the Biological Effects of PM_2.5_ from Greater Cairo

**DOI:** 10.3390/ijms20204970

**Published:** 2019-10-09

**Authors:** Sara Marchetti, Salwa K. Hassan, Waleed H. Shetaya, Asmaa El-Mekawy, Elham F. Mohamed, Atef M. F. Mohammed, Ahmed A. El-Abssawy, Rossella Bengalli, Anita Colombo, Maurizio Gualtieri, Paride Mantecca

**Affiliations:** 1 Department of Earth and Environmental Sciences, Research Center POLARIS, University of Milano-Bicocca, 20126 Milan, Italy; s.marchetti16@campus.unimib.it (S.M.); rossella.bengalli@unimib.it (R.B.); anita.colombo@unimib.it (A.C.); 2 Air Pollution Research Department, Environmental Research Division, National Research Centre, 33 El-Bohouth St., Dokki, Giza 12622, Egypt; salwakamal1999@gmail.com (S.K.H.); wh.shetaya@nrc.sci.eg (W.H.S.); asmaamekawy75@gmail.com (A.E.-M.); elham_farouk0000@yahoo.com (E.F.M.); ateffathy2006@yahoo.com (A.M.F.M.); elabssawysalem@gmail.com (A.A.E.-A.); 3 ENEA SSPT-MET-INAT Bologna Research Centre; Via Martiri di Monte Sole, 4; 40129 Bologna, Italy; maurizio.gualtieri@enea.it

**Keywords:** air pollution, PM_2.5_, toxicity, lung, in vitro systems

## Abstract

Greater Cairo (Egypt) is a megalopolis where the studies of the air pollution events are of extremely high relevance, for the geographical-climatological aspects, the anthropogenic emissions and the health impact. While preliminary studies on the particulate matter (PM) chemical composition in Greater Cairo have been performed, no data are yet available on the PM’s toxicity. In this work, the in vitro toxicity of the fine PM (PM_2.5_) sampled in an urban area of Greater Cairo during 2017–2018 was studied. The PM_2.5_ samples collected during spring, summer, autumn and winter were preliminary characterized to determine the concentrations of ionic species, elements and organic PM (Polycyclic Aromatic Hydrocarbons, PAHs). After particle extraction from filters, the cytotoxic and pro-inflammatory effects were evaluated in human lung A549 cells. The results showed that particles collected during the colder seasons mainly induced the xenobiotic metabolizing system and the consequent antioxidant and pro-inflammatory cytokine release responses. Biological events positively correlated to PAHs and metals representative of a combustion-derived pollution. PM_2.5_ from the warmer seasons displayed a direct effect on cell cycle progression, suggesting possible genotoxic effects. In conclusion, a correlation between the biological effects and PM_2.5_ physico-chemical properties in the area of study might be useful for planning future strategies aiming to improve air quality and lower health hazards.

## 1. Introduction

The World Health Organization (WHO) stated that air pollution is the major environmental and public health risk, due to its effect on global air quality and climate. The WHO reported that, indeed, each year, about 7 million people die because of air pollution, with 4.2 million of these deaths (60%) caused by the exposure to outdoor ambient air pollution [1]. For that reason, greater attention should be payed to environmental pollution and its effects on human health, possibly by planning and implementing national, long-term health policies. Cooperation with non-governmental organizations is also necessary to identify and try to mitigate the several environmental threats and sources of air pollutants. Planning and control programs to manage air quality should be implemented to reduce air pollution and the associated adverse health effects, and finally, obtain an improvement in public health all over the world [2,3].

In recent years, many developing countries, like Egypt, have experienced rapid urbanization, industrial development, construction activities and an increase in traffic flow, all of which seriously threaten the environment with the huge amounts of pollutants emitted. One of the major air pollutants is particulate matter (PM), which contributes to acidification of precipitation and climatic change, and affects Earth’s radiation balance, agriculture, material and cultural heritages and ecosystems [4,5,6]—but is also one of the most deleterious pollutants for human health.

Acute and long-term exposure to PM has been associated with an increase in mortality and morbidity in the population [2,3]. Several studies in the last decades have associated particulate exposure to premature deaths, allergies [4], ischemic heart disease or strokes, respiratory illness and infections [2], chronic obstructive pulmonary disease (COPD), exacerbation of asthma and increased cancer incidence, especially lung cancer [7]. Nevertheless, the mechanisms of action involved in the pathogenesis of such adverse health effects are still debated and different PM physicochemical properties, such as size, surface area and chemical composition, are suggested to be determinants for their onset [8]. 

PM is a complex and heterogeneous mixture of natural and anthropogenic origin composed of water-soluble inorganic compounds, organic carbons, and elemental carbon and metals. It is emitted from direct sources, and/or formed from conversion of gases into secondary particles through chemical reactions [9]. The differences in emission sources and the subsequent atmospheric chemical reactions lead to increase in variation in size, shape, surface area, chemical composition, solubility and origin of the aerosol in the atmosphere [10,11].

Studies have been performed in Greater Cairo (Egypt) on urban, residential, suburban and industrial areas reporting the organic PM (Polycyclic Aromatic Hydrocarbons, PAHs) and inorganic (i.e., sulphate and nitrate) PM’s chemical compositions, including the volatile organic compounds (VOCs), potentially toxic trace and major elements and gases [12,13,14]. These studies evidenced high levels of atmospheric pollution, suggesting that low air quality should be considered the most serious environmental concern in Greater Cairo. The general climate of Cairo city is cold, moist and rainy in winter, whereas it is characterized by high temperatures, high solar radiation, a clear sky and being rainless, during summer. Cairo may be also exposed to three types of episodes: dust storms (during spring), haze dust and straw rice combustions (during autumn).

However, the knowledge is limited on the levels of fine PM (PM_2.5_) in Greater Cairo, as is the case in regard to its chemical composition, its biological impact and its possible health effects. Our previous work [15] reported that the average concentrations of PM_2.5_ in the Dokki urban area were higher than the maximum yearly standards in the European Commission’s environmental quality standards for ambient air (25 mg/m^3^). PM_2.5_ concentrations also exceeded the Egyptian air quality standard for PM_2.5_ value (80 µg/m^3^ for 24 h) during December 2010 [16].

PM_2.5_ pollution in fact greatly varies at the regional scale, depending on the local emission sources and climatic and geographic variables. These parameters have been shown to affect the biological reactivity and thus the final impact on human health [17]. 

Different toxicological mechanisms have been suggested to be activated by airborne particles. Several studies on in vitro systems reported the release of pro-inflammatory mediators, oxidative stress [18], cell death [19] and genotoxicity [20] as the main effects observed after exposure to PM. Cell cycle alterations are also reported [21]. Furthermore, accumulating evidence demonstrates a strong seasonality of the adverse health effects produced by PM, with differences according to climatic conditions and emission sources [4,22]. In vivo studies also demonstrated a link between PM exposure and lung inflammation, which triggers the onset of cardiovascular adverse events [23,24].

At present, no literature is available on the toxicological profile of PM from Greater Cairo, despite the great importance attributable to the knowledge of the specific toxic outcomes of the particles sampled in specific local representative sites. To fill this gap of knowledge, the present work aimed at comparatively investigating the chemical composition and in vitro toxic effects of the urban PM_2.5_ collected during different seasons in the urban area of Dokki, Giza (Greater Cairo). 

After samplings, PM_2.5_ was analyzed for ionic species, elements and PAHs, while the toxicity was evaluated on the human alveolar epithelial cells, A549. The cytotoxic, inflammatory and genotoxic potentials of the PM_2.5_ samples collected during spring, summer, autumn and winter were investigated.

In addition to the basic knowledge on the toxicity mechanisms associated to the Greater Cairo urban PM_2.5_, these results will be relevant for implementing future risk mitigation strategies, not only on the basis of PM mass concentration, but also considering the specific PM biological effects. 

## 2. Results

### 2.1. PM_2.5_ Physico-Chemical Characterization 

The average seasonal variations of the concentrations (mg/g) of the water-soluble ionic species in PM_2.5_ during the period of study at the Dokki urban area are shown in Table 1. From that table, is evident that sulphate and nitrate were the ions with the highest concentrations, whereas the lowest levels were found for Mg^2+^ and K^+^ during the four seasons. The highest concentration of water-soluble ions was detected in summer. Statistical analysis revealed significant differences among seasons, showing a higher content of ionic species in summer compared to spring (*p* < 0.0001), autumn (*p* < 0.001) and winter (*p* < 0.0001). Among all the water-soluble ions determined in the present study, SO_4_^2−^ was the most abundant PM_2.5_ chemical component of the different seasons. The total secondary inorganic ions (NO_3_^−^, SO_4_^2−^ and NH_4_^+^) were the dominant water-soluble ions in PM_2.5_ during the four seasons.

Table 2 summarizes the seasonal variations in the metal/metalloid contents (mg/g) of the PM_2.5_ samples. It is evident that Al and Fe were the dominant elements during the four seasons, followed by Pb, Cu, Zn and Mn. The minimum concentrations were noted for Co and Hg. The concentrations of the individual measured metals were found to follow the following pattern: Al > Fe > Pb > Cu > Zn > Mn > V > Ni > As > Cd > Co > Hg. The highest mean concentrations of the measured metal were found in spring, whereas the lowest levels were detected in summer. Significant differences were observed between seasons, with an increase in metal content during spring in comparison to summer (*p* < 0.001) and autumn (*p* < 0.0001). Winter was found statistical augmented with respect to autumn (*p* < 0.001). These results indicate that crustal metals (Al and Fe) were the most abundant constituents in PM_2.5_ of the study area.

The mean seasonal concentrations (mg/g) of the individual PAH compounds during the period of study are shown in Table 3. It can be noticed that BGP, DBA, IND, BaP, BkF, BbF and CRY were the most abundant PAH compounds during the different seasons. NA and ACY were the lowest concentrations during the period of study. These results also indicate that the concentrations of PAHs were similar in winter and summer. In addition, statistical differences revealed that PAHs’ content was increased in summer compared to spring (*p* < 0.001) and autumn (*p* < 0.05). Significant differences were observed also between winter and spring (*p* < 0.0001).

The apportionment of PAHs according to selected, characteristic ratio values (Table S1) shows that the main difference between summer–spring and winter–autumn seasons is related to the contribution of coal and biomass burning emission. This source can explain also the differences observed in PM atmospheric concentration. 

**Figure 1:**
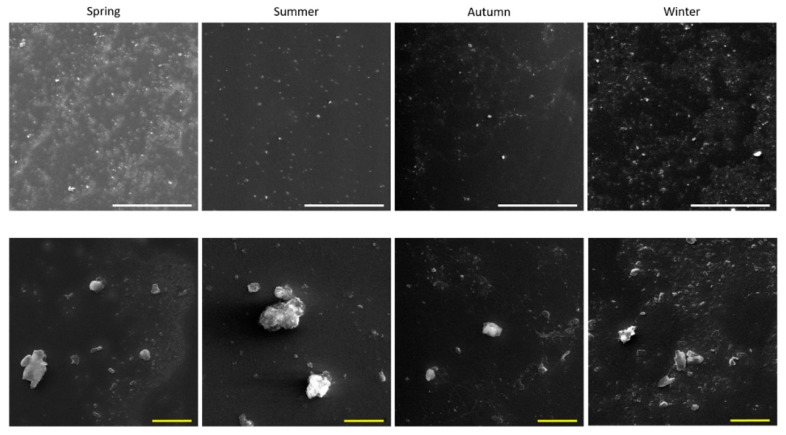
PM_2.5_ morphological characterization. Scanning electron microscope observations on the extracted particles: spring, summer, autumn and winter. Particles were observed to the concentration of 25 μg/mL. White scale bar: 100 μm. Yellow scale bar: 10 μm.

The morphological characterization of PM_2.5_ obtained by scanning electron microscope analysis is shown in Figure 1. According to their respective sampling season, the particles that seems to be well dispersed have a size of less than 10 µm. 

The endotoxin levels in PM-extracted samples are reported in the Supplementary Materials (Figure S1). The results showed that all PM_2.5_ samples contained about 60 EU/mL, with no significant seasonal variations. 

### 2.2. Biological Responses 

#### 2.2.1. Cell Viability 

**Figure 2:**
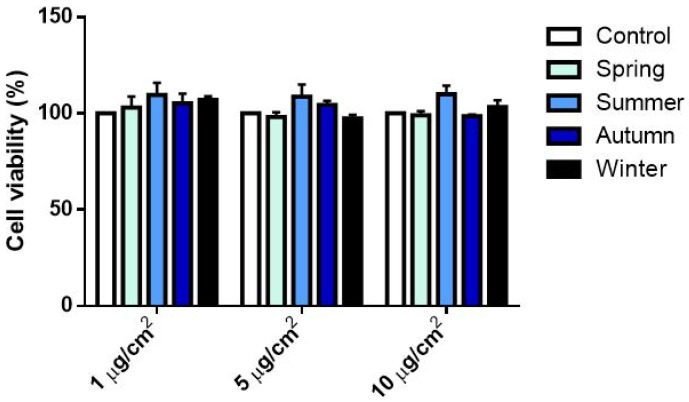
Cellular metabolic activity assessed by Alamar Blue assay after 24 h exposure to increasing doses (1, 5 and 10 µg/cm^2^) of PM_2.5_ collected in different seasons. Each bar shows mean ± SEM of four independent experiments (*n* = 4). Statistical differences among seasons were analyzed by two-way ANOVA with Dunnett’s multiple comparisons test.

The impact of PM_2.5_ samples on cell viability was assessed by Alamar Blue assay after exposure to increasing doses (1–10 µg/cm^2^). None of the treatments produced any significant alterations in cellular metabolic activity when compared to the corresponding control (Figure 2).

#### 2.2.2. Release of Inflammatory Mediators

**Figure 3:**
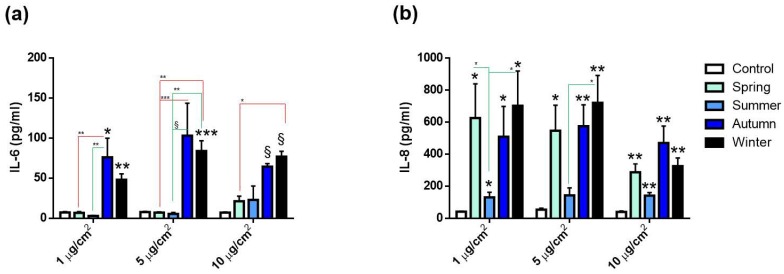
Pro-inflammatory cytokines’ releases. IL-6 (**a**) and IL-8 (**b**) protein secretion by A549 cells after 24 h of exposure to increasing doses (1, 5 and 10 µg/cm^2^) of PM_2.5_ collected in different seasons. Each bar shows mean ± SEM of four independent experiments (*n* = 4). PM versus control. Statistical analysis was performed by unpaired *t*-test. Statistical differences among seasons were analyzed by two-way ANOVA with Tukey’s multiple comparisons test. ^§^ *p* < 0.0001, *** *p* < 0.001, ** *p* < 0.01 and * *p* < 0.05.

The activation of the inflammatory response was evaluated by measuring the secretions of the cytokines IL-6 and IL-8 after 24 h exposure to all the PM_2.5_ doses tested. IL-6 release significantly increased only after exposure to autumn and winter PMs (Figure 3a). Spring and summer PMs did not affect IL-6 levels. On the other hand, IL-8 secretion was significantly increased after exposure to all the PMs, although to a lesser extent, following treatment with the one collected during summer (Figure 3b). Interestingly, significant differences were observed among seasons: autumn and winter PMs indeed induced an increased release of IL-6 compared to spring and summer PMs. Regarding IL-8 instead, differences among seasons were observed only between summer and autumn, with the latter statistically increased compared to the warmer season..

#### 2.2.3. Cell Cycle Alterations 

**Figure 4:**
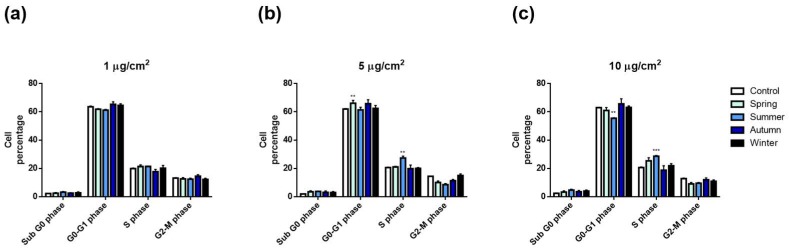
Cell cycle analysis of A549 cells after 24 h exposure to increasing doses (1, 5 and 10 µg/cm^2^—(**a**,**b**,**c**), respectively) of PM_2.5_ collected in different seasons. Each bar shows mean ± SEM of four independent experiments (*n* = 4). Statistical analysis was performed by two-way ANOVA with Dunnett’s multiple comparison test. *** *p* < 0.001 and ** *p* < 0.01 versus control cells.

The effects on the cell cycle progression after exposure to the PM_2.5_ from the different seasons was also evaluated. Spring and summer PMs seemed to affect DNA replication, since a significant increase in S-phase cell populations and a parallel decrease of those in G2/M phase was observed (Figure 4c). On the contrary, the exposure to autumn and winter PMs did not induce cell cycle alterations at any of the tested doses (Figure 4a–c). 

#### 2.2.4. Oxidative Stress and PAH Metabolizing Enzymes’ Activations

**Figure 5:**
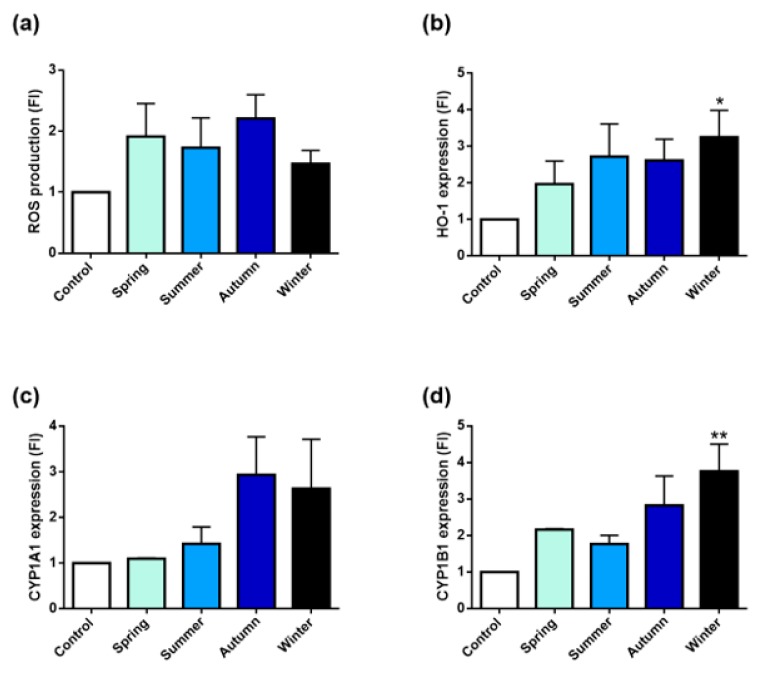
Oxidative stress and xenobiotics’ metabolism pathways’ activations in A549 cells after exposure to 10 µg/cm^2^ seasonal PM_2.5_. (**a**) Intracellular reactive oxygen species (ROS) generation induced by the different PMs after 3 h of treatment. (**b**) HO-1 protein expression in A549 upon exposure to PM_2.5_ for 24 h. CYP1A1 (**c**) and CYP1B1 (**d**) protein expression after 24 h of treatment. Bars represent means ± SEM of three independent experiments (*n* = 3). Statistical analysis was performed by one-way ANOVA with Dunn’s (**b**) and Dunnett’s (**c**,**d**) multiple comparison test. ** *p* < 0.01 and * *p* < 0.05 versus control cells.

Oxidative stress and xenobiotics’ metabolism responses were investigated following exposure to 10 µg/cm^2^ PM_2.5_ for 24 h. Since it represents an early cell response, the reactive oxygen species (ROS) production was measured after 3 h exposure (Figure 5). 

As shown in Figure 5a, PM samples induced a slight increase of intracellular ROS in respect to control, but no significant differences among the sampling seasons existed.

To determine whether the exposure to the different PMs triggered an anti-oxidant response, the expression of the stress-inducible heme-oxygenase 1 protein (HO-1) was also evaluated. As shown in Figure 5b, HO-1 expression was increased after exposure to all PMs, but a significantly higher value was registered only for winter PM. 

The induction of CYP1A1 and CYP1B1 expression was also measured (Figure 5c,d). As indicated in Figure 5c, PM_2.5_ collected during spring and summer had a very limited effect on CYP1A1 expression. Instead, autumn and winter induced CYP1A1 more efficiently. A similar trend was observed also for the induction of CYP1B1. The latter protein indeed, was found to be increased by all PMs, with winter PM inducing the highest and most statistically significant effect (Figure 5d). 

### 2.3. Correlations between Chemical Parameters and Biological Effects

**Figure 6:**
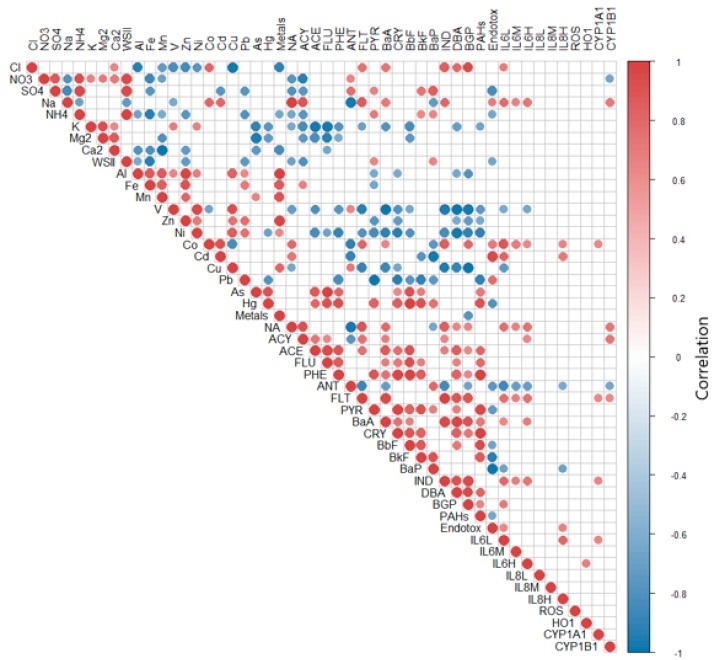
Correlation diagram of chemical species and biological responses analyzed from fine PM extracted from filters sampled during the different seasons. Dark red spots indicate high positive correlation while dark blue ones indicate negative correlation. Only the correlations with statistical significance *p* < 0.01 are reported. Number of variables = 50; number of samples = 16 (the chemical parameters are considered constant during each season according to the pooling of homogeneous filters).

Overall correlation showed expectedly high, positive correlations within the compounds belonging to the same chemical class; namely, metals and trace elements, PAHs and soluble ions (Figure 6).

The figure reports only the correlations that showed significant (*p* < 0.01) positive or negative correlations; Fe, Mn, Zn, Al and Ni correlated among themselves and with the total sum of metals and trace elements (Metals); similarly, sulphates (SO_4_^2^^−^), nitrate (NO^3^^−^) and ammonia (NH_4_^+^) correlated among each other and with the sum of the water-soluble inorganic ions (WSIs). Of major interest, the correlation graph showed correlations, mainly positive, between the proteins analyzed and the chemical properties of the sampled PMs. Of interest, Cyp enzymes correlated positively with organic compounds (NA, ACY, FLT and IND), providing evidence of the activation of gene transcription in response to PM-bounded PAHs. Interesting is the correlation with cobalt (Co), whose emission in the atmosphere is related to combustion of coal and other human activities. Accordingly, the source’s contribution to the characteristic PAHs ratio (Table S2) supports an increase of coal combustion during autumn and winter.

**Figure 7:**
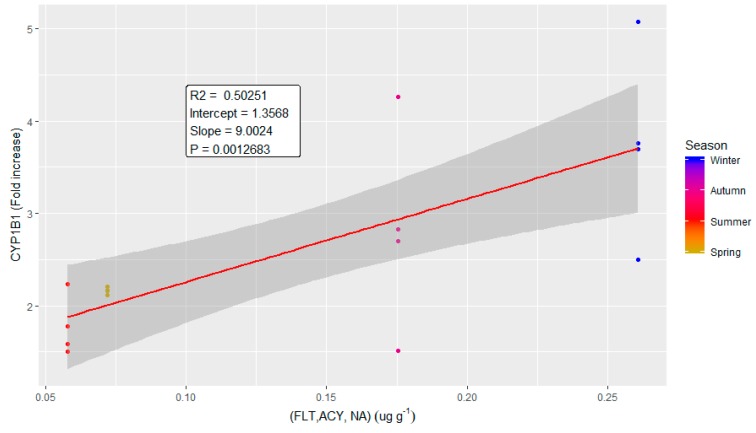
Correlation plot of the modulation of CYP1B1 protein expression and a linear correlation of the chemical variables identified from the correlation diagram reported in Figure 6. NA, FLT and ACY were linearly combined to obtain a single variable describing the variation of these compounds along the sampling campaign. The data are reported in different colours for the different seasons. The linear correlation (in red) is reported with its 95% confidence interval (grey shadow). The parameters describing the correlation curve are also reported in the plot.

The correlation plot of CYP1B1 (Figure 7) with the selected compounds (FLT, ACY and NA) showed R^2^ equal to 0.50, a slope of 1.36 and a *p*-value of 0.001. Despite the limited correlation value, which was, however, expected—due the complexity of the biological responses to air pollution and the interplay of different biological pathways within the cells—the plot is statistically significant and clearly shows an increase of the response moving from summer samples to winter ones. 

A similar correlation plot was obtained for CYP1A1 and a linear combination of organic compounds (FLT and IND) and cobalt. For that protein, the plot values were: R^2^ =0.39, slope = 1.5 and *p*-value = 0.005. 

Among the inflammatory proteins, interesting correlations were determined (the respective plots are reported in the supplementary materials: Figures S3, S4 and S5), and in general showed a lower contribution to the biological effects of PM sampled in spring, and a higher contribution to winter samples. IL-6, for the high dose of treatment (IL6H), is correlated to organic (FLT, NA, IND, BaA) and inorganic (Na and Co) compounds (Supplementary Figure S2), while IL-6 at the medium dose of treatment (IL6M) corelates to Na, Co, FLT, NA and IND (Figure S3). Correlations were also found between IL-8 (at the high dose of treatment, IL8H) and Cd and endotoxin (Figure S4). Interestingly, the correlation of IL-8 with endotoxin and Cd contributed differently in the four seasonal samples: summer PM had the weakest association, while autumn PM the strongest. Considering the two variables independently (Figures S4A and S4B), it appears that the correlation of IL-8 with endotoxin was driven mainly by autumn and spring PM samples, rather than from winter and summer ones. This peculiar correlation was likely due to the relationship of airborne bacterial distribution and meteorological parameters, such as temperature and relative humidity. 

For HO-1, IL-8 (at low and medium doses of treatment) and IL-6 (at the lower dose of treatment), the correlation plots were not significant (*p*-value > 0.05) and are not reported.

## 3. Discussion

Recently, great attention has been devoted to air pollution in developing countries, like Egypt and sub-Saharan countries. In this research effort, seasonal PM_2.5_ was, for the first time for the region, collected to evaluate and correlate its physical, chemical and biological properties. PM_2.5_ samples were collected in the Greater Cairo region, where urbanization and industrialization have rapidly increased in the last few decades, causing an increase in the atmosphere’s pollution [25]. The high rate of emission from industrial activities, electric power stations and traffic density, coupled with low wind speeds and the frequent thermal inversions in the area, resulted in high local pollution load. Therefore, Greater Cairo is considered one of the most polluted megacities in the world, as reported by several works. Boman et al. [15] in 2013, investigated the PM_2.5_ mass concentration in a urban residential area, Dokki, the same as our study. They stated that between September 2010 and May 2011, the PM_2.5_ concentration was 51 µg/m^3^, well above the EU and WHO quality standards set for health protection. Krzyzanowski instead, in 2014, reported the cities in the world with the highest PM_2.5_ concentrations. Among those, the annual mean reported in Cairo was 80 µg/m^3^, one of the highest observed [26]. Those data have been confirmed more recently by Shaltout [27]. 

Previous studies performed in Greater Cairo area were focused only on the seasonal differences in chemical composition, areal distributions and concentrations of airborne particles. Studies performed by Boman [15] and Shaltouth [27] reported higher concentrations of PM_2.5_ during winter months and related that seasonality to the lower height of the boundary layer and lower wind speed in winter, which leads to reducing the dispersion of particulate matter in the air. The relative peak of PM_2.5_ concentrations observed during the spring months, may be an effect of the hot “Khamsin” southerly wind, which occurs in Egypt, predominantly during this season, and which bring air masses loaded with dust and sand [15]. Hassan [12] also reported that during autumn, winter and spring, several types of pollution episodes, including straw rice combustions, haze dust and dust storms, could represent a severe environmental hazard in Egypt. 

Nevertheless, the biological effects of such particles collected in Greater Cairo are still unknown. Therefore, the main aim of our study was to define the cytotoxic effects produced on lung epithelial cells by PM_2.5_ collected in Giza during the different seasons and investigate the relationship between the adverse effects observed and the particulate’s chemical composition. In the present study, PM_2.5_ samples were analyzed for PAHs, metals and water-soluble inorganic ions (WSIs). Furthermore, cytotoxic, pro-inflammatory and genotoxic effects were evaluated in vitro on human A549 alveolar cells. 

Regarding the chemical composition, water soluble ions were the most representative compound, with seasonal differences observed mainly in the SO_4_^2−^ concentration. WSIs predominantly were found in the summer PM_2.5_, due to the high temperatures and photochemical reactions [28,29]. PM_2.5_ was characterized also by a high PAH content, especially in summer and winter, compared to spring and autumn. Among metals, the higher amount was observed in spring and the lowest in autumn, with Al and Fe being the most abundant metals in all the seasons.

Furthermore, morphological characterization showed that, once extracted from the filters, PM_2.5_ samples were mainly constituted by irregularly shaped particles, mostly in the fine range, which tended to form aggregates. The content of endotoxins in Giza PMs was also evaluated. No significant differences according to the sampling season were observed. On the contrary, previous works performed in different regions reported a higher endotoxin content in PM samples collected during summer [22,30]. These results, however, are not surprising, considering that the endotoxin content can vary among the different seasons and cities and is influenced by environmental factors, like humidity and temperature [31,32].

The PM_2.5_-induced biological effects were analyzed according to the sampling season by evaluating cell viability, inflammatory response and cell cycle progression in human lung A549 cells exposed for 24 h to PM doses representative of a daily particle deposition on a lung. Literature data on highly polluted regions reported that PM_2.5_ concentrations can exceed 100 μg/m^3^/24 h [33,34]. Those concentrations correspond to 0.06 and 3 μg/cm^2^ deposited in the alveolar and tracheobronchial regions, respectively [33]. The selected doses were, therefore, in accordance with those reported by Li et al. [33], according to which the in vitro dose range of 0.2–20 μg/cm^2^ was necessary to observe biological effects. Biomarkers of cell oxidative stress and the xenobiotic metabolizing system were also evaluated. 

No effects on cell viability were detected after exposing cells at the selected PM_2.5_ doses (Figure 2). Accordingly, previous data on PM_2.5_ biological responses have shown no significant effects on cell viability [29,35]. Herein, we report a significant release of inflammatory mediators (IL-6 and IL-8) after fine PM exposure. The inflammatory response is, therefore, related to the specific chemical composition of particles [36], as also showed in Figure 6 and in Supplementary Materials. 

Nevertheless, considering that endotoxins are known inducers of inflammation, and given their high content in all samples, we would have expected a strong inflammatory response after exposure to all the seasons’ samples investigated, and possibly in a dose-response manner. Conversely, not all samples induced cytokine production on a linear dose-response manner. A slight modulation of the cytokines’ release was indeed observed at the highest dose, especially for IL-8, suggesting a possible hormetic effect, in which the biological system adapts responses to environmental insults by improving its functionality and tolerance. Another possible explanation for that reduction might be the possible alteration of interleukins’ secretion by PM_2.5_ exposure. A recent study indeed showed that PM_2.5_, at 10 µg/cm^2^, increased mRNA-synthesis and intracellular protein levels of IL-6 and IL-8, but it appeared to impair cytokine release in bronchial cells, in particular that of IL-8, possibly by altering cytoskeletal organization involved in protein secretion [35]. The individual composition of the tested PMs could also lead to different cell inflammatory responses, as already demonstrated in literature [30,37].

The main results point out that the toxicity responses were dependent upon the seasonal PM_2.5_’s chemical variability. Regarding the release of pro-inflammatory cytokines, only the PM_2.5_ collected in autumn and winter induced an increase in IL-6 secretion, while IL-8 was increased after exposure to all PMs (Figure 3). 

At a low concentration exposure, it was not possible to define a significant correlation between PM chemical composition and IL’s release. On the contrary, the correlations between IL-6 and the mean and high doses of treatment, and IL-8 with the higher dose of treatment, revealed interesting associations. IL-6 was related at both the mean and high doses of treatment to the presence of naphthalene (NA), anthracene (ANT), indeno(1, 2, 3,-cd)pyrene (IND), cobalt (Co) and sodium (Na) suggesting the importance of both combustion and natural sources in determining the increase of inflammatory responses. However, with exposure to the high concentration, other compounds (namely, ACY, BaA and Vanadium) were associated to an increased protein release. This difference seems to suggest that (i) besides the combustion process other anthropogenic sources as well, likely industrial ones, may contribute to the inflammatory potency of PM, and (ii) that when increasing the doses, the complexity of the biological response pathways may cover or uncover the importance of specific chemical species. For IL-8, a significant correlation was found between Cd and endotoxin. Again, this specific correlation may signify that this protein responds more rapidly to the bacterial compounds, well known to induce inflammatory responses, but that the release is highly affected by other compounds, such as Cd, that can modulate the intensity of the response. 

Autumn and winter PMs were also able to increase the CYP1A1 and CYP1B1 expressions, and HO-1’s expression was also significantly higher after winter PM_2.5_ exposure. The increase in CYP enzymes was positively related to specific organic species, again suggesting the importance of combustion activities emitting those compounds. We reported an interesting association between CYP1A1 with PAHs and Co. Since the combustion of coal and some industrial activities may determine the contemporaneous emission of these compounds, it will be relevant in future studies to evaluate the contribution of these sources to local air quality. However, it is important to remember the differential behavior of individual PAHs when combined in a mixture, in relation to biological responses. Individual PAHs indeed, are able to modulate CYP1A1 and CYP1B1 genes in different ways; however, their effect when combined in a mixture of compounds is uncertain [38,39]. 

According to these observations, in the human alveolar A549 cells, the PM_2.5_ collected during the colder seasons mainly induced the xenobiotic metabolizing system and the consequent antioxidant and pro-inflammatory cytokine release responses, which can be interpreted as active defense mechanisms. Under the conditions tested, in fact, neither the cell viability nor the cell cycle progression were affected after exposure to autumn and winter PM_2.5_. In the winter PM_2.5_, the highest amount of Fe was also measured (Table 2). This transition metal may have had contributed to the increased intracellular red-ox activity, which finally resulted in HO-1 overexpression, although no significant correlations were found. 

Our results are consistent with literature data reporting the release of pro-inflammatory cytokines in epithelial cells exposed to PM with a high PAH content [39,40]. Moreover, they are in agreement with previous observations where particles collected in different seasons induced variable biological effects [41,42,43]. 

Regarding the effects of the PMs collected during the warmer seasons, only summer PM_2.5_ induced a concentration-dependent arrest in S-phase, with a corresponding decrease in the percentage of G0/G1 cells (Figure 4). This points out that-phase arrest is related to altered DNA replication, suggesting the presence of DNA damage or an alteration of the DNA replication machinery. In the summer PM_2.5_, the highest concentrations of ionic species, especially sulphates, and a particularly high concentration of the genotoxic PAH, BaP, were measured (Table 1 and Table 2). Deng et al. [44] supported our results by showing that inorganic extracts from dust storm and PM_2.5_ can alter cell cycle progression in human lung fibroblasts, inducing accumulations of cells arrested in S-phase. Organic extracts instead, induced alterations in G0–G1 phase [44]]. It had already been shown that nitro-PAHs and BaP alter cell cycle progression in different human cell lines, inducing S-phase arrest [45,46]. In particular, Hockley [46] reported *S*-phase accumulation in human hepatocarcinoma (HepG2) after exposure to BaP. All considered, we can conclude that the summer PM_2.5_, enriched in sulphates and BaP, may pose a genotoxic risk. It is interesting to note that, in parallel to the genotoxic insult, there was no activation of the defense mechanisms, such as cytokine secretion and CYP-mediated metabolism (Figure 3 and Figure 5). 

It is well known that inflammatory response and oxidative stress play a key role in the PM-induced toxic effects [2]. A slight increase in ROS generation was observed for the sampled PMs, with no significant differences among the seasons. The results were confirmed by western blot analysis, in which an increased expression in HO-1 was observed, especially after exposure to winter PM, suggesting the activation of the anti-oxidant response. HO-1 indeed, is a defense protein induced in several cell types, both in vivo and in vitro, in the presence of oxidative stress and inflammatory stimuli [24,47]. Our results are in agreement with literature’s data, showing the modulation of the oxidative stress response as a common mechanism triggered by exposure to fine PM [48,49].

## 4. Materials and Methods 

### 4.1. Site Description and PM_2.5_ Collection 

**Figure 8:**
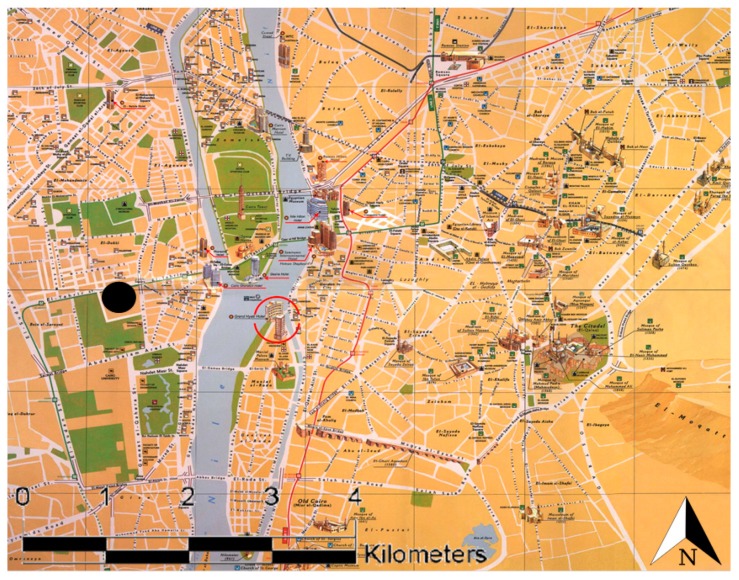
Detailed map of the Greater Cairo area, showing Cairo city with its streets (white lines), the Nile river (blue area) and several parks (green areas). Black dot represents our sampling site in Dokki district.

The Greater Cairo region (Cairo, Giza, and Shoubra El-Khiema cities) lies to the south of Delta in the Nile basin. The narrow strip of Giza Governorate runs along the western side of the River Nile, opposite to the city of Cairo (Figure 8). It lies between two big industrial areas, one in the north (Shoubra El-Khiema) and the other in the southeast (Helwan).

Sampling collection was carried out from December 2016 to November 2017. Samples were collected from a point approximately 25 m above the ground level on the roof of the National Research Centre (NRC). The NRC is in the urban area of Giza (Dokki, 30°2′10.148″ N, 31°12′0.419″ E), situated to the southwest of Cairo’s city center (Figure 1), which is characterized by high traffic density. Daily (24 h, two samples per week; 24 samples per season) PM_2.5_ samples were collected using a both high-volume air sampler for analysis of the chemical composition and a low volume Dewell–Higgins type cyclone sampler (Casella CEL, Bedford, UK) for the biological effects. 

PM_2.5_ samples for chemical analysis were collected on glass fiber filters (20.3 cm × 25.4 cm) using the high-volume sampler. Glass fiber samples were equilibrated for 24 h, using controlled temperature and relative humidity desiccators, before and after sampling. The collected PM_2.5_ mass was determined by the difference in weight of the filter’s mass before and after sampling; then, PM_2.5_ concentration was calculated from the mass and volume of air. After weighting, filters were stored at 4 °C until chemical analysis, to prevent evaporation of volatilized components. For the analysis, each filter was cut into four parts and one piece for each filter was used for ionic species, metals or PAHs’ detection respectively. Filters collected during the same season were pooled to obtain data representative of the entire sampling season. 

A low volume Dewell–Higgins type cyclone sampler (Casella CEL, Bedford, UK) with a Teflon filter (25 mm, 0.2 µm) was used to collect PM_2.5_ for biological investigations. Blank control filters were collected from all sampling campaigns and treated similarly to the others.

### 4.2. PM_2.5_ Chemical Characterization 

#### 4.2.1. Water Soluble Inorganic Ionic Species (WSIs)

One quarter of each filter was cut into pieces and the water-soluble components were extracted into 20 mL of distilled water in 50 mL polypropylene tubes by ultrasonic bath (KQ300DE, Kunshan Ultrasonic Instrument Co., Ltd., China) for 40 min. The water samples were filtered with a 0.45 μm PTFE syringe filter (Pall Co. Ltd., USA) and stored at 4 °C until analysis. An ion chromatography system (Thermo IC-5000) was used to analyze the concentrations of three anions (Cl^−^, NO_3_^−^ and SO_4_^2−^) and five cation (Ca^2+^, Mg^2+^, NH_4_^+^, K^+^ and Na^+^) species. The anions were detected with an Ion Pac ASRS-4 suppressor and an Ion Pac AS11-HC × 250 mm analytical column. The eluent for anion analysis was 10 mmol/L Na_2_CO_3_. The cations were detected with an Ion Pac CSRS-4 suppressor and an Ion Pac CS12A × 250 mm analytical column. The eluent for cations analysis was 10 mmol/L CH_4_O_3_S. Injection of the samples was done automatically using a 10 μL loop. External standard solutions were used to generate the standard curve, and the correlation coefficient was higher than 0.999. Replicates and blanks were checked every 10 sample runs for quality control. Blank samples are analyzed in the same way to evaluate analytical bias and precision with ion chromatography. All the blanks were lower than method detection limits (MDLs), and the concentration data for the WSII were corrected by these filter blanks. The MDLs were calculated as three times the standard deviations of seven replicate blank samples. The MDLs for SO_4_^2−^, NO_3_^−^, Cl^−^, NH_4_^+^, K^+^, Na^+^, Ca^2+^ and Mg^2+^ were 0.001 μg/m^3^.

#### 4.2.2. Elemental Concentration

Filters and blanks were extracted by acid digestion. Filters were cut into small pieces, placed in a conical flask and added with 1 M nitric acid at 70 °C for 3 h in ultrasonic bath and analyzed by inductively coupled plasma optical emission spectrometry (ICP MS) to determine the concentrations of toxic metals/metalloids (Al, Zn, Fe, V, Mn, Co, Ni, Cu, As, Pb, Hg and Cd). Multi-element analysis was undertaken using an Agilent 8800 Triple Quadrupole ICP-MS (ICP-QQQ) operated with helium in the collision cell to eliminate isobaric interferences. The ICP-QQQ was equipped with a standard sample introduction system, including a glass concentric nebulizer, quartz spray chamber and torch, and nickel-tipped cones. Samples and calibration standards were introduced from an Agilent ASX-500 Series auto-sampler. Internal standards, including Rh and Ir (10 µg/L) were directly introduced to the sample stream via a T-piece. For Hg measurements, internal standards were 10 µg/L In and Rh prepared in a matrix of ultra-pure 1% HNO_3_/0.5% HCl. To reduce memory effects and improve the washout of Hg from the ICP system a washing protocol including (a) 1 g/L EDTA, 0.08 g/L Triton X-100 and 6 g/L NH_4_OH, (b) technical grade 5% HNO_3_/5% HCl and (c) ultra-pure 1% HNO_3_/0.5% HCl, was introduced between samples [50]. Limits of detection (LOD) was calculated from 16 blanks and all analyses were run in triplicate.

#### 4.2.3. Polycyclic Aromatic Hydrocarbons (PAHs)

PAHs were extracted in a Soxhlet apparatus for 24 h using purified normal hexane and dichloromethane (DCM) mixture (50/50, *v*/*v*) according to Fang et al. [51]. The organic extracts were then concentrated using a rotary evaporator, cleaned by clean silica gel/alumina columns consisting of 5 g anhydrous sodium sulfate, 20 g silica gel (deactivated 5% with distilled water), 10 g alumina (deactivated 1% with distilled water), 5 g sand, and glass wool according to Park et al. [52]. The extracts were concentrated, exchanged to 2 mL hexane, placed on the columns, and eluted with 1:1 pentane–dichloromethane (200 mL). The eluted extracts were concentrated on a rotary evaporator and exchanged to 1 mL hexane and stored in a freezer until analysis. For PAHs analysis, a 1-μL extract was withdrawn from the samples, including the blank samples, and injected into a Hewlett-Packard gas chromatography (GC; model HP6890), fitted with a flame ionization detector. A HP-5 (30 m × 320 μm × 0.25 μm) capillary column was used with hydrogen as carrier gas. The concentrations of the target PAH compounds were quantified by an external standard solution of 15 PAH compounds (PAH mixture, Supelco, Inc., Cairo, Egypt). The concentrations of the following PAH compounds in the particulate phase were determined: naphthalene (NA), acenaphthylene (ACY), acenaphthene (ACE), fluorene (FLU), phenanthrene (PHE), anthracene (ANT), fluoranthene (FLT), pyrene (PYR), benzo(a)anthracene (BaA), chrysene (CRY), benzo(b)fluoranthene (BbF), benzo(a)pyrene (BaP), dibenzo(a,h)anthracene (DBA), benzo(ghi)perylene (BGP) and indeno (1, 2, 3,-cd)pyrene (IND).

### 4.3. Filter Extraction and Particle Characterization

For in vitro toxicity studies, filters from the same season were extracted in water by mechanical detachment, together with the adsorbed compounds. To maximize particle extraction and recovery, we used also an ultrasound bath (Sonica Soltec, Milan, Italy) by replicating five 20-min ultrasonic cycles. Particle suspensions were then collected in sterile tubes previously weighed, and dried into a desiccator [29]. Desiccation process was performed using a dryer put under vacuum at room temperature (RT) in the dark with warm silica gels for about two weeks (until complete water evaporation). Silica gels were substituted, and vacuum restored every two days. Once dried, the sterile tubes were weighted to determine the mass of the extracted particles and stored at −20 °C until further use. This procedure assured a good efficiency of extraction, guaranteeing the similarity of the extracted particles to the collected ones [53].

For cell exposure, particles were suspended in sterile water to obtain aliquots at a final concentration of 2 µg/µL. PMs were sonicated for 30 secs just prior to cell exposure. 

Particles extracted from filters and re-suspended in water were morphologically characterized by scanning electron microscopy. Briefly, aliquots of 8 µL of sonicated particle suspensions at 25 μg/mL in pure water and 1% Isopropyl alcohol were respectively, pipetted on aluminum stubs and dried at RT. Samples were then graphite-coated and observed by a Tescan VEGA 5136XM, operating at 20 kV. Images were digitally acquired and elaborated through dedicated software.

The presence of endotoxins in the extracted PM_2.5_ was assessed using the Limulus Amebocyte Lysate (LAL) chromogenic quantitative detection kit (GenScript, PiscatawayNJ, USA), according to manufacturer’s instructions.

Briefly, PM extracts for cell treatments at the concentration of 2 µg/µL were diluted with endotoxin-free LAL reagent water to the concentration of 10 µg/mL, mixed with the LAL supplied in the kit and incubated at 37 °C for 16 min. A chromogenic substrate solution was then mixed with samples and incubated at 37 °C for additional 6 min. The reaction was stopped with three-color stabilizers. The absorbance of each reaction was read by a multiplate spectrophotometer reader (Tecan, Männedorf, Switzerland) at 545 nm. The endotoxin content of each sample was calculated from a standard curve of *Escherichia coli* and the concentration expressed as endotoxin units per milligram (EU/mg) of tested particles.

### 4.4. PM_2.5_ In Vitro Toxicity 

#### 4.4.1. Cells Culture and Exposure Conditions 

PM biological effects were evaluated on A549 cells, a human lung adenocarcinoma epithelial cell line obtained from the American Type Culture Collection (CCL-185, American Type Culture Collection, Manassas, VA, USA). Cells were cultured at 37 °C in a humidified atmosphere containing 5% CO_2_ in OptiMEM I Reduced Serum Medium (Gibco, Life Technologies, Monza, Italy) supplemented with 10% heat-inactivated fetal bovine serum (FBS, Gibco) and 1% Penicillin/Streptomycin (100 X, Euroclone, Pero, Italy).

Cells were seeded 24 h before treatment at a concentration of 2 × 10^4^ cells/cm^2^ in 12-well plates or 1.6 × 10^4^ cells/cm^2^ in 6-well plates. Culture medium was removed and replaced by 1% FBS supplemented OptiMEM medium and cells exposed for 24 h at 37 °C to increasing PM_2.5_ concentrations (1, 5 and 10 µg/cm^2^) in 12-well plates for cell viability, inflammatory response and cell cycle alteration evaluations. Cells exposed to extracts from blank filters were also used as additional negative control (see Supplementary Materials, Figure S6).

For oxidative stress investigations and protein expression analysis cell were exposed to 10 μg/cm^2^ PM in 6-well plates. Unexposed cells (treated with cellular medium only) were used as control.

#### 4.4.2. Cell Viability Assay 

Cell viability was measured in A549 cells exposed to PM_2.5_ (1–10 µg/cm^2^) for 24 h by means of the Alamar Blue assay (Invitrogen, Burlington, ON, Canada). Briefly, at the end of the exposure, medium was collected and stored for inflammatory response evaluation (more detail in the following paragraph), cells were rinsed with phosphate buffer saline (PBS) and incubated for 3 h in a solution containing 1:10 of Alamar Blue reagent and OptiMEM complete medium. The absorbance of each sample, proportional to cell viability, was measured by a multiplate spectrophotometer reader (Tecan, Männedorf, Switzerland) at excitation and emission wavelengths of 570 and 630 nm, respectively. Cell viability was calculated as a percentage of viable cells with respect to control (unexposed) samples.

#### 4.4.3. Pro-Inflammatory Cytokine Release

At the end of treatments, cell culture supernatants were recovered and centrifuged at 12,000 rpm for 6 min at 4 °C to remove debris and floating cells. The resulting supernatants were collected and stored at −20°C until the test was performed. IL-6 and IL-8 protein levels were measured by sandwich ELISA, according to the manufacturer guidelines (Life Technologies, Monza, Italy). The absorbance of each sample, whose color intensity is proportional to the amount of cytokine, was measured at 450 nm and 630 nm. The amount of proteins in pg/mL was calculated based on a standard curve.

#### 4.4.4. Flow Cytometry Analysis of the Cell Cycle

Cell cycle distribution after exposure to PM_2.5_ was assessed by DNA-staining with propidium iodide (PI). Briefly, after 24 h of exposure, cells were trypsinized, harvested, fixed in ice cold 90% EtOH and stored at −20 °C until analysis. Then, cell suspensions were centrifuged (1600 rpm for 6 min), the supernatants discarded, and the pellet incubated in PBS and RNAse DNAse-free (20 µg/mL, Sigma-Aldrich, Saint Louis, MO, USA Italy) for 30 min at 37 °C. Finally, DNA was stained with the fluorescent dye PI (10 μg/mL, Sigma Aldrich, Milan, Italy) and 10,000 cells per sample were scanned by flow cytometry, using 617 nm band pass filter (CytoFLEX 13/3, Beckman Coulter, Krefeld, Germany). The different phases of the cell cycle were assessed with CytExpert analysis software (Krefeld, Germany).

#### 4.4.5. ROS Production 

The intracellular production of reactive oxygen species (ROS) was analyzed by flow cytometry. A549 cells were plated in 12-well plates at a density of 2 × 10^4^ cells/cm^2^ and allowed to grow for 24 h. A549 cells were incubated with 5 μM Carboxy-2′,7′-Dichlorofluorescein Diacetate (carboxy-DCFDA, 2 mM, Life Technologies, Monza, Italy) in PBS for 20 min at 37 °C. Cells were then washed in PBS and exposed to 10 μg/cm^2^ PM for 3 h. At the end of incubation, cells were trypsinized, harvested and suspended in PBS. The ROS production, detectable by the oxidation of the probe, was quantified by measuring the fluorescence intensity in the FITC channel of 10000 events with the cytometer CytoFLEX (Beckman Coulter, Krefeld, Germany). Data were analyzed using the CytoExpert software.

#### 4.4.6. Western Blot Analysis

After 24 h of exposure to 10 μg/cm^2^ PM in 6-well plates, cells were scraped and lysed on ice with RIPA buffer (150 mM NaCl, 1% Triton X-100, 0.5% sodium deoxycholate, 0.1% SDS, 50 mM Tris pH 8.0) and 0.1% of proteases inhibitor, added just before use. Cellular lysates were centrifuged at 12000 rpm for 15 min and collected to determine the protein concentration by bicinchoninic acid assay (Sigma Aldrich, Milan, Italy), performed according to the manufacturer instructions. 30 µg of proteins were loaded onto 12% SDS-PAGE gels, separated and transferred on nitrocellulose membranes. Blocking buffer in Tris-Buffered Saline (TBS) with 0.1% Tween20 (TBS-T) supplemented with 5% w/v bovine serum albumin (BSA; Sigma) or milk (Skim milk powder, Fluka, Sigma, Milan, Italy) was added to incubate the membranes for 1 h. After blocking, membranes were incubated in rabbit polyclonal antibodies HO-1 (1:1000, Cell Signaling Technology, Danvers, CO, US), CYP1A1 and CYP1B1 (1:500, Novus Biologicals, Littleton, CO, United States) O/N at 4 °C. The membranes were then washed three times with TBS-T and incubated in Blocking buffer for 1 h at RT with the specific HRP-linked secondary antibodies (anti-rabbit IgG, 1:2000, Cell Signaling). Membranes were finally washed and detected by enhanced chemiluminescence (ECL, Euroclone). Digital images were taken by a luminescence reader (Biospectrum-UVP, LLC, Upland, CA, United States) and densitometry analysis performed with dedicated software (Vision Works LS, Cambridge, UK). Monoclonal anti-β-Actin antibody (Cell Signaling, 1:1000) was used as loading control.

### 4.5. Statistical Analyses

Data on chemical characterization are expressed as mean ± standard deviation between individual samples of the same season. 

Data on biological responses are expressed as the mean ± standard error of mean (SEM) of at least three biological replicates carried out following the same experimental conditions. For the chemical and biological endpoints, the statistical analyses were performed by unpaired *t*-test, one-way or two-way ANOVA with Dunn’s, Dunnett’s or Tukey’s post hoc multiple comparisons tests, using the software Graph Pad, Prism Program version 6. Values of *p* < 0.05 were considered statistically significant.

Correlations among the characterized chemical parameters and the biological effects, obtained on the pooled PM samples, were evaluated by the software environment R Studio (RStudio Team, 2016, Boston, Massachusetts, USA) running on R version 3.5.1 (R Core Team, 2018 University of Auckland, New Zealand). General correlations were determined (Hmisc, package, Frank E Harrell Jr, with contributions from Charles Dupont and many others, 2019, Nashville, Tennessee, USA) among selected biological parameters; namely, IL6, IL8, CYP1B1, CYP1A1 and HO-1, and the chemical variables analysed. Where different concentrations of exposure were performed, the data from each dose were maintained (such as for interleukins) in order to investigate also possible chemistry dose-related effects. The final matrix was composed by 50 columns (40 chemical plus 10 biological variables) and 16 rows (the chemical variables were replicated during each season while the biological responses from each experiment were reported). From the results obtained, specific correlation plots were performed between a single biological output and one or more relevant chemical variables previously identified. If different compounds were selected for the correlation plot, their linear combination were used in order to weight each variable equally. The overall correlation plot reports only the correlations that were significant (*p* < 0.01) while for the single linear correlation plot the R^2^, the line coefficient and the *p*-value of the correlation are reported. 

## 5. Conclusions

Our data demonstrate that the toxic potential of the urban PM_2.5_ from Greater Cairo is linked to the seasonal changes in the chemical composition. The variable content in PAHs, metals and ionic species, indeed determined different biological effects in human lung cells exposed in vitro. 

The fine PMs from colder seasons promoted cell xenobiotic responses and highly pro-inflammatory effects, while the ones from warmer seasons displayed a direct effect on cell cycle progression. These results were significantly associated to specific organic and inorganic species and are in line with previous studies reporting PM-induced toxic effects on lung cells, associated to season-related changes in composition [29,41,54]. Of course, the seasonal-dependent biological effects are affected by the regional climatological variables and emission sources, which finally determine a great variability in the physico-chemical characteristics of the PM pollution. Considering the developing countries, improvements in the characterizations of the emission sources, by means of source apportionment techniques, in parallel to the studies on the PM biological reactivity and epidemiological evidences, are highly recommended to better characterize the potential health risks and possible future mitigation strategies.

## Figures and Tables

**Table 1:** Contents of water-soluble inorganic ions (WSII) in fine particulate matter (PM_2.5_) collected during 2017 (mg/g).

Water-Soluble Inorganic Ions Title	Spring	Summer	Autumn	Winter
Cl^−^	22.66 ± 4.57	33.79 ± 1.54	42.67 ± 25.99	33.71 ± 10.94
NO_3_^−^	50.81 ± 11.24	65.42 ± 15.14	55.58 ± 3.64	38.35 ± 14.02
SO_4_^2−^	106.74 ± 21.52	204.33 ± 24.44	107.02 ± 8.10	86.13 ± 20.29
Na^+^	23.94 ± 10.52	19.83 ± 1.88	30.73 ± 19.39	32.93 ± 11.01
NH_4_^+^	28.63 ± 1.97	53.33 ± 2.01	37.86 ± 0.79	25.60 ± 10.33
K^+^	20.67 ± 1.16	18.41 ± 6.09	19.83 ± 2.45	13.02 ± 6.44
Mg^2+^	19.16 ± 5.48	18.55 ± 2.41	21.40 ± 9.22	13.59 ± 8.49
Ca^2+^	33.96 ± 3.18	35.75 ± 5.28	37.99 ± 19.59	32.47 ± 10.86
Ʃ WSIs	306.58 ^§^ ± 59.65	449.42 ± 58.79	353.08 ^***^ ± 89.17	275.79 ^§^^‡^ ± 92.37

Seasonal mean concentrations (mg/g) of water-soluble inorganic ions (WSII) in PM_2.5_ sampled by a high-volume sampler in Dokki urban area during the period of study. ± Standard deviation between individual samples of the same season. *n* = 24 for each season. Statistical differences among seasons were analyzed by one-way ANOVA with Tukey’s multiple comparisons test. ^§^ *p* < 0.0001, ^***^ *p* < 0.001 summer compared to other seasons, ^‡^ *p* < 0.01 autumn versus winter.

**Table 2:** Contents of potentially toxic metals/metalloids in PM_2.5_ collected during 2017 (mg/g).

Metals	Spring	Summer	Autumn	Winter
Al	32.41 ± 8.26	23.11 ± 5.08	20.93 ± 0.15	27.98 ± 7.86
Fe	14.4 ± 7.34	8.58 ± 2.32	9.96 ± 0.13	14.54 ± 4.5
Mn	0.50 ± 0.08	0.44 ± 0.10	0.39 ± 0.02	0.52 ± 0.09
V	0.31 ± 0.11	0.23 ± 0.07	0.20 ± 0.01	0.18 ± 0.04
Zn	0.99 ± 0.61	0.68 ± 0.15	0.69 ± 0.01	0.86 ± 0.17
Ni	0.31 ± 0.10	0.19 ± 0.03	0.20 ± 0.02	0.17 ± 0.02
Co	0.011 ± 0.00	0.010 ± 0.00	0.053 ± 0.01	0.033 ± 0.03
Cd	0.09 ± 0.08	0.02 ± 0.00	0.18 ± 0.01	0.11 ± 0.09
Cu	1.27 ± 0.06	0.99 ± 0.30	0.75 ± 0.01	0.88 ± 0.14
Pb	2.93 ± 0.61	2.08 ± 1.13	2.60 ± 0.23	2.5 ± 0.78
As	0.16 ± 0.03	0.18 ± 0.05	0.15 ± 0.01	0.2 ± 0.06
Hg	0.008 ± 0.00	0.010 ± 0.00	0.008 ± 0.00	0.010 ± 0.00
Ʃ Ms	53.39 ± 15.68	40.51 ^***^ ± 6.24	36.12 ^§^ ± 0.41	47.96 ^‡^ ± 12.40

Seasonal mean concentrations (mg/g) of potentially toxic metals/metalloids (Ms) in PM_2.5_ collected during the period of study. ± Standard deviation between individual samples of the same season. *n* = 24 for each season. Statistical differences among seasons were analyzed by one-way ANOVA with Tukey’s multiple comparisons test. ^§^ *p* < 0.0001 and ^***^ *p* < 0.001 spring versus summer and autumn, ^‡^ *p* < 0.001 autumn versus winter.

**Table 3:** Contents of organic PM (Polycyclic Aromatic Hydrocarbons, PAHs) in PM_2.5_ collected during 2017 (mg/g).

PAHs	Spring	Summer	Autumn	Winter
NA	0.17 ± 0.09	0.13 ± 0.03	0.28 ± 0.04	0.33 ± 0.01
ACY	0.25 ± 0.02	0.23 ± 0.06	0.26 ± 0.05	0.31 ± 0.01
ACE	0.32 ± 0.02	0.42 ± 0.10	0.38 ± 0.06	0.53 ± 0.01
FLU	0.29 ± 0.02	0.38 ± 0.09	0.29 ± 0.04	0.47 ± 0.01
PHE	0.46 ± 0.03	0.60 ± 0.14	0.52 ± 0.11	0.61 ± 0.01
ANT	1.84 ± 0.10	2.31 ± 0.38	0.52 ± 0.10	0.63 ± 0.02
FLT	1.69 ± 0.08	1.93 ± 0.38	2.41 ± 0.42	2.55 ± 0.04
PYR	1.85 ± 0.10	2.17 ± 0.48	1.99 ± 0.37	2.05 ± 0.03
BaA	2.10 ± 0.13	2.29 ± 0.50	2.38 ± 0.44	2.49 ± 0.02
CRY	3.42 ± 0.15	4.21 ± 0.89	3.87 ± 0.59	4.10 ± 0.04
BbF	4.27 ± 0.25	4.95 ± 1.01	4.41 ± 0.62	5.06 ± 0.09
BkF	3.71 ± 0.17	5.13 ± 1.04	3.74 ± 0.57	4.48 ± 0.04
BaP	3.21 ± 0.19	4.12 ± 0.71	2.78 ± 0.36	3.31 ± 0.06
IND	2.33 ± 0.13	2.79 ± 0.54	3.49 ± 0.56	3.50 ± 0.05
DBA	3.25 ± 0.23	3.84 ± 0.77	3.94 ± 0.77	4.12 ± 0.06
BGP	3.64 ± 0.20	4.35 ± 0.78	4.78 ± 0.70	4.62 ± 0.06
ƩPAHs	32.82 ± 1.90	39.85 ^§^ ± 7.20	36.05 ^‡^ ± 5.78	39.14 ^§^ ± 0.55

Seasonal mean concentrations (mg/g) of PAHs in PM_2.5_ sampled by a high-volume sampler in the Dokki urban area during the period of study. ± Standard deviation between individual samples of the same season. *n* = 24 for each season. Statistical differences among seasons were analyzed by one-way ANOVA with Tukey’s multiple comparisons test. ^§^ *p* < 0.0001 spring versus summer and winter, ^‡^ *p* < 0.05 summer versus autumn.

## Supplementary Materials

Supplementary materials can be found at https://www.mdpi.com/1422-0067/20/20/4970/s1.

## Abbreviations

WHO	World Health Organization
PM	particulate matter
COPD	chronic obstructive pulmonary disease
PAHs	polycyclic aromatic hydrocarbons
VOCs	volatile organic compounds
NA	Naphthalene
ACY	Acenaphthylene
ACE	Acenaphthene
FLU	Fluorene
PHE	Phenanthrene
ANT	Anthracene
FLT	Fluoranthene
PYR	Pyrene
BaA	Benzo(a)anthracene
CRY	Chrysene
BbF	Benzo(b)Flouranthene
BkF	Benzo(k)Flouranthene
BaP	Benzo (a) pyrene
IND	Indeno(1,2,3-cd) pyrene
DBA	Dibenzo(a,h) anthracene
BGP	Benzo(ghi)perylene
WSIs	water-soluble ions
Ms	metals/metalloids
MDLs	method detection limits
ICP MS	Inductively Coupled Plasma Optical Emission Spectrometry
ICP-QQQ	Triple Quadrupole ICP-MS
DCM	dichloromethane
GC	gas chromatography
LAL	Limulus Amebocyte Lysate
PI	Propidium Iodide
ROS	reactive oxygen species
H_2_O_2_	Hydrogen peroxide
TBS	Tris-Buffered Saline
TBS-T	TBS with 0.1% Tween20
BSA	bovine serum albumin
