# Supplementary material for: Seasonal Variation in the Biological Effects of PM2.5 from Greater Cairo
Source: Int J Mol Sci. 2019 Oct 9;20(20):4970. doi: 10.3390/ijms20204970 (PMC6829270; doi:10.3390/ijms20204970)
Supplement: Supplementary file 1 [file ijms-20-04970-s001.pdf]

Supplementary materials

Seasonal variation in the biological effects of $PM_{2.5}$ from Greater Cairo

Sara Marchetti[1], Salwa K. Hassan[2], Waleed H. Shetaya[2], Asmaa El-Mekawy[2], Elham F. Mohamed[2], Atef M. F. Mohammed[2], Ahmed A. El-Abssawy[2], Rossella Bengalli[1], Anita Colombo[1], Maurizio Gualtieri[3], Paride Mantecca[1*]

[1] University of Milano-Bicocca, Department of Earth and Environmental Sciences, Research Center POLARIS, Milan, Italy; s.marchetti16@campus.unimib.it; rossella.bengalli@unimib.it; anita.colombo@unimib.it; paride.mantecca@unimib.it

[2] Air Pollution Research Department, Environmental Research Division, National Research Centre, 33 El-Bohouth St., Dokki, Giza 12622, Egypt; salwakamal1999@gmail.com; wh.shetaya@nrc.sci.eg; asmaamekawy75@gmail.com; elham_farouk0000@yahoo.com; ateffathy2006@yahoo.com; elabssawysalem@gmail.com;

[3] ENEA SSPT-MET-INAT Centro Ricerche Bologna; Via Martiri di Monte Sole, 4; 40129 Bologna BO, Italy; maurizio.gualtieri@enea.it

* Correspondence: paride.mantecca@unimib.it; Tel.: +39- 0264482916

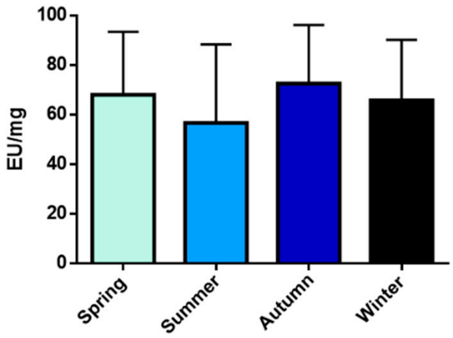

Figure S1. Endotoxin content of seasonal $PM_{2.5}$ samples determined by LAL test. Data are mean ± SEM of three independent experiments (N = 3). Statistical differences among seasons were analysed by One-Way ANOVA with Tukey's multiple comparisons test.

Correlations between chemical parameters and biological effects

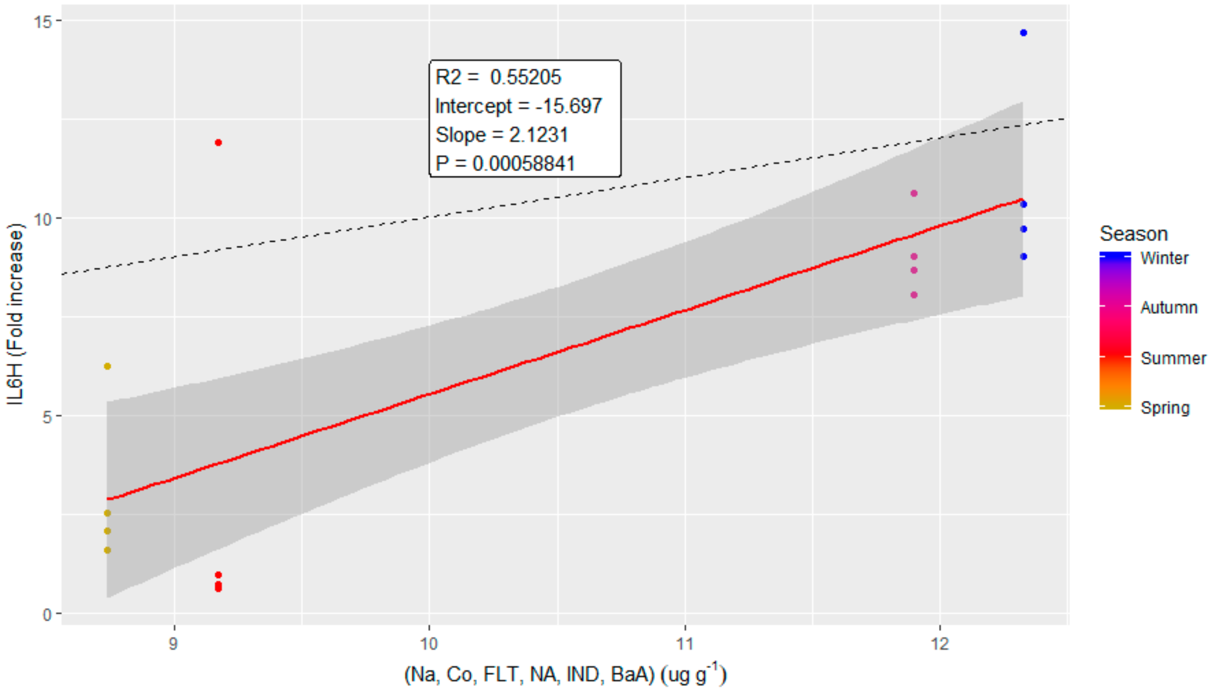

Figure S2. Correlation plot of the modulation of IL6H protein expression and a linear correlation of the chemical variables identified from the correlation diagram reported in Figure 6. The data are reported in different colours for the different seasons. The linear correlation (in red) is reported with its 95% confidence interval (grey shadow). The parameters describing the correlation curve are also reported in the plot.

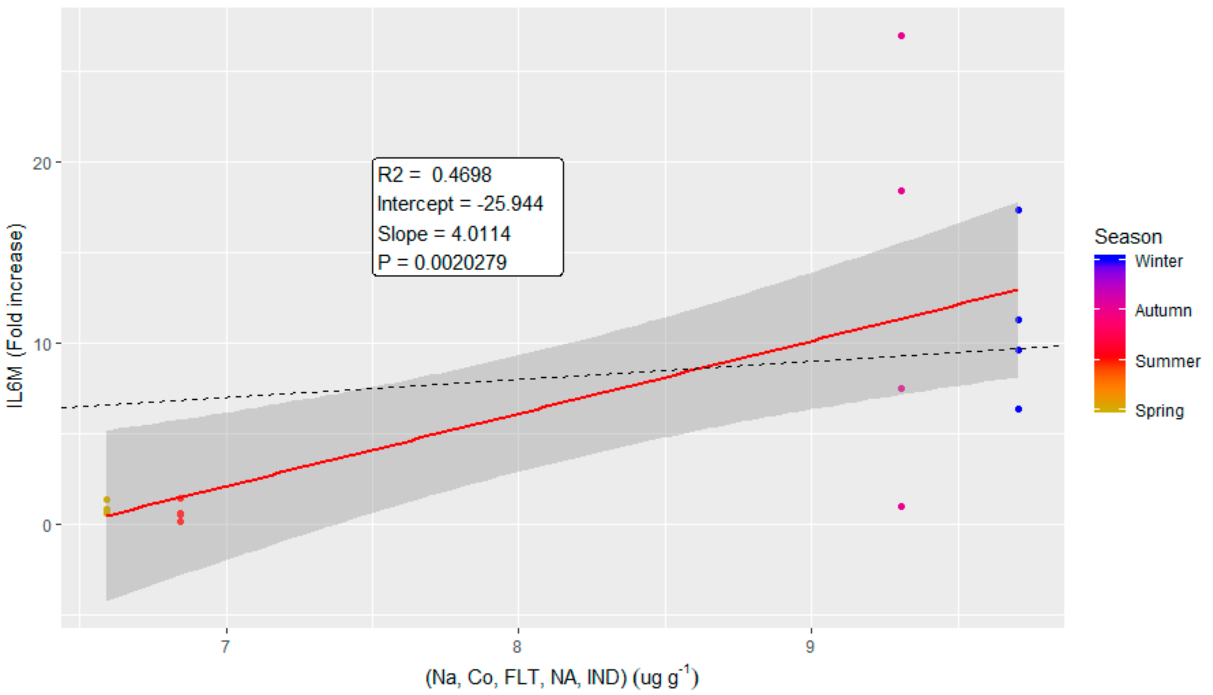

Figure S3. Correlation plot of the modulation of IL6M protein expression and a linear correlation of the chemical variables identified from the correlation diagram reported in Figure 6.

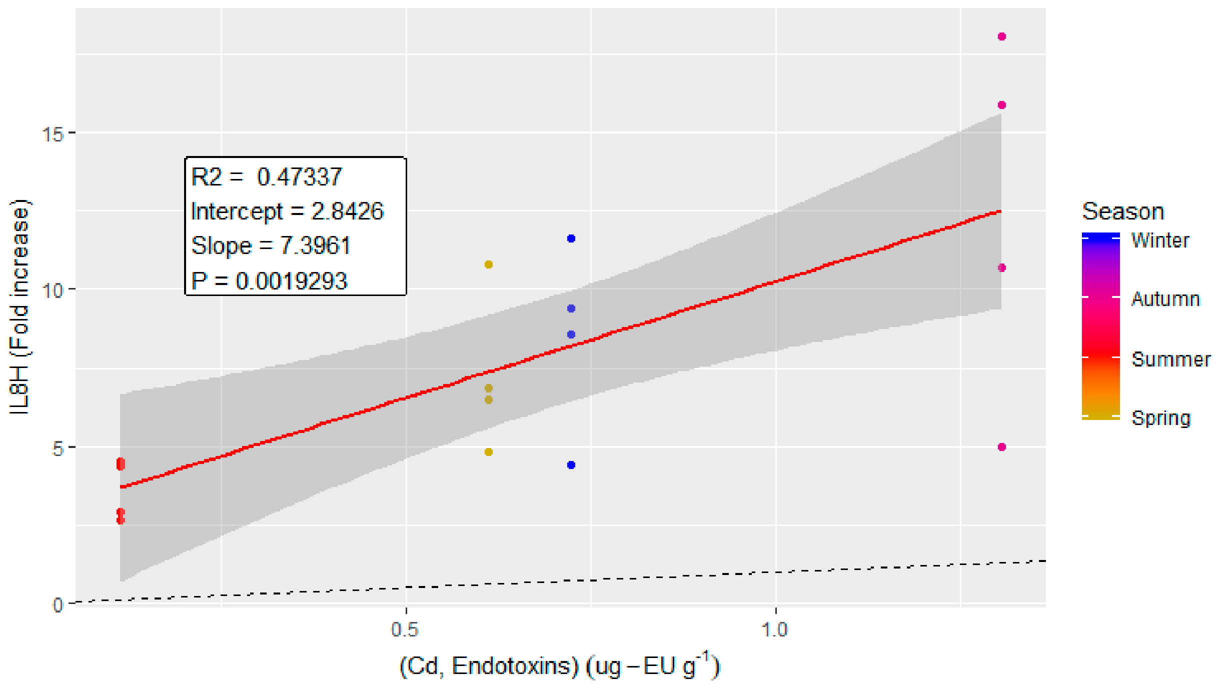

Figure S4. Correlation plot of the modulation of IL8H protein expression and a linear correlation of fine PM endotoxin and cadmium content as relevant chemical parameter identified from the correlation diagram reported in Figure 6.

A

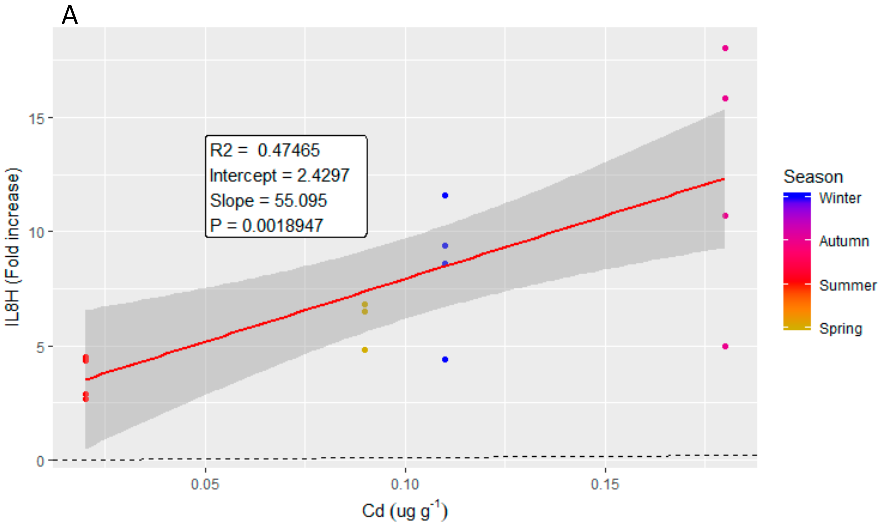

B

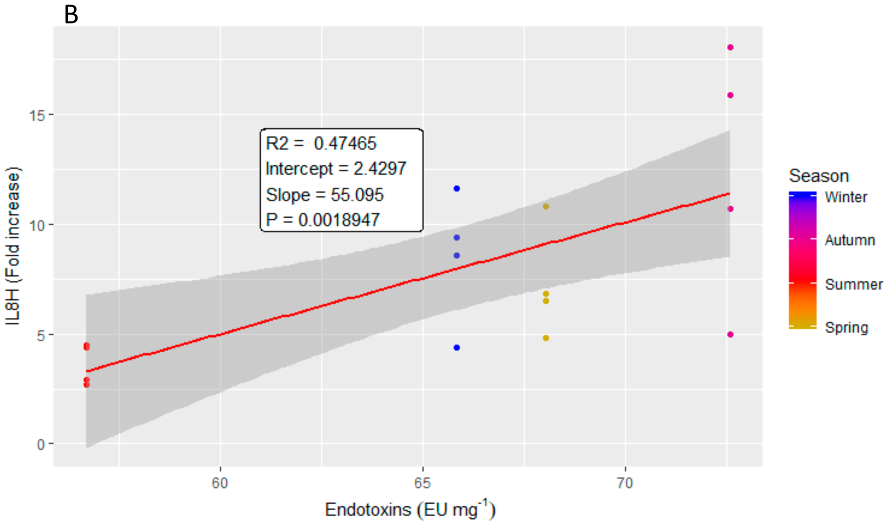

Figure S5. Correlation plots of the modulation of IL8H protein expression and a linear correlation of Cd (A) and Endotoxin (B).

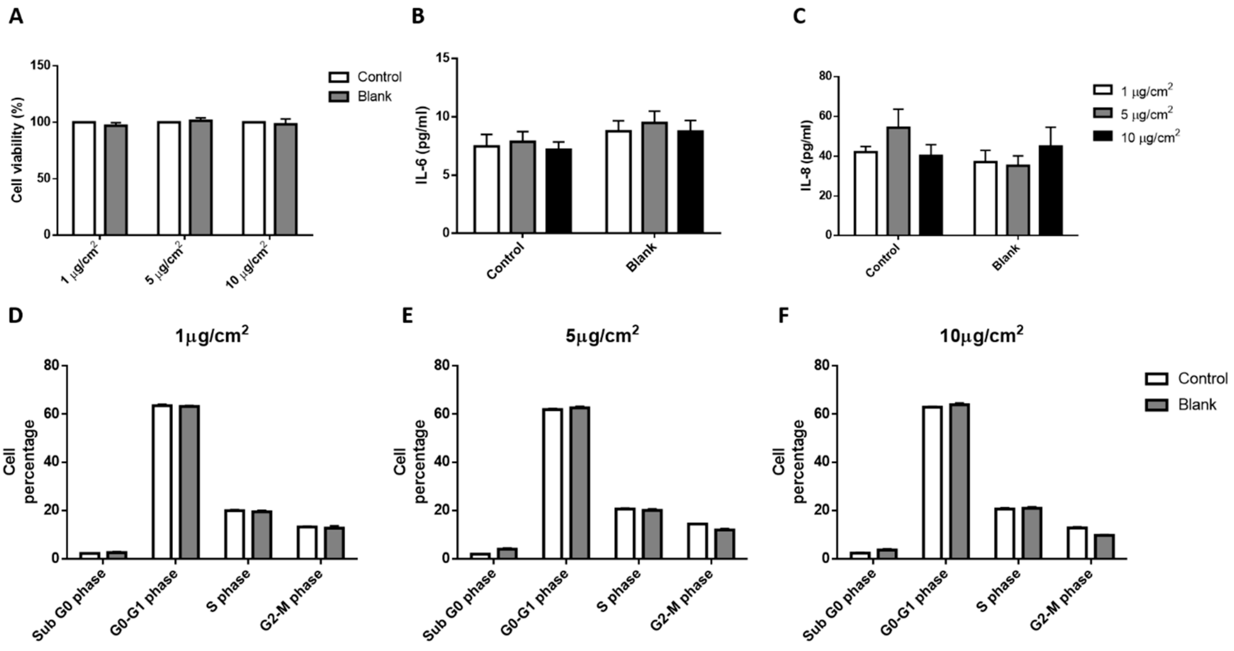

Figure S6. Control cells vs. cells exposed to extracts from blank filters doses (1, 5 and 10 μg/cm²). Viability (A), cytokine release (B, C) and cell cycle analysis (D, E, F). Each bar shows mean ± SEM of four independent experiments (N=4). Statistical differences among seasons were analyzed by Two-Way ANOVA with Sidak's multiple comparisons test.

Table S1. Source identification of PAHs emissions by means of characteristic ratio values

**Diagnostic ratio**	**Spring**	**Summer**	**Autumn**	**Winter**	***Characteristic ratio values***		
IND/(IND+BGP)[a]	0,39	0,39	0,42	0,43	*0.35-0.70 Diesel burning*	*0.18 Cars*	*0.62 Biomass burning*
FLU/(FLU+PYR)[a]	0,14	0,15	0,13	0,19	*< 0.5 Gasoline*	*> 0.5 Diesel*	
BAP/(BAP+CRY)[b]	0,48	0,49	0,42	0,45	*> 0.35 Vehicular emission*	*0.2-0.35 Coal Combustion*	
BAP/BGP[a]	0,88	0,95	0,58	0,72	*0.5 - 0.6 Traffic emission*	*> 1.25 Brown coal or lignite*	
ANT/(ANT+PHE)[b]	0,80	0,79	0,50	0,51	*> 0.1 pyrogenic*	*< 0.1 petrogenic*	
IND/BGP[a]	0,64	0,64	0,73	0,76	*< 0.4 [illegible]ine*	*1 Diesel*	
FLT/(FLT+PYR)[b]	0,48	0,47	0,55	0,55	*< 0.4 petrogenic*	*0.4 – 0.5 fossil fuels*	*> 0.5 Grass, wood, coal combustion*
FLT/PYR[b, c]	0,91	0,89	1,21	1,24	*< 0.6 non traffic emissions*	*> 0.6 traffic emissions*	*1.0-1.4 Coal combustion*

a = Ravindra et al., 2008, b = Tobiszewski and Namiesnik., 2012, c = Lee et al., 1982

The results from characteristic ratio values show that during winter and autumns there is an increase in wood and coal combustion in relation to local domestic and industrial activities. This ratio (FLT/(FLT+PYR)) seems the most representative of the differences between the seasons of PAHs concentration. High differences are related to FLT/PYR ratio which also confirms that during winter coal combustion is a significant emitter of airborne fine PM.

References

Lee, L.M., Vassilaros, L.M. and Douglasr, W.L. 1982. Capillary Column Gas Chromatography of Environmental Polycyclic Aromatic Compounds. Int. J Environ. Anal. Chem. 11: 251–262

Ravindra, K., Sokhi, R., Van Grieken, R. 2008. Atmospheric polycyclic aromatic hydrocarbons: source attribution, emission factors and regulation. Atmos. Environ. 42, 2895–2921.

Tobiszewski M. and Namiesnik J. 2012. PAH diagnostic ratios for the identification of pollution emission sources Environmental Pollution 162, 110-119
